# Direction-of-Arrival (DoA) Estimation Performance for Satellite Applications in a Multipath Environment with Rician Fading and Spatial Correlation

**DOI:** 10.3390/s23125458

**Published:** 2023-06-09

**Authors:** Mutmainnah Hasib, Sithamparanathan Kandeepan, Wayne S. T. Rowe, Akram Al-Hourani

**Affiliations:** School of Engineering, Royal Melbourne Institute of Technology (RMIT) University, Melbourne 3000, Australia; kandeepan.sithamparanathan@rmit.edu.au (S.K.); wayne.rowe@rmit.edu.au (W.S.T.R.); akram.hourani@rmit.edu.au (A.A.-H.)

**Keywords:** direction-of-arrival (DoA) estimation, geostationary Earth orbit (GEO), low Earth orbit (LEO), multipath environment, Rician fading, spatial correlation

## Abstract

Direction-of-arrival (DoA) estimation methods are highly versatile and find extensive applications in satellite communication. DoA methods are employed across a range of orbits, from low Earth orbits (LEO) to geostationary Earth orbits (GEO). They serve multiple applications, including altitude determination, geolocation and estimation accuracy, target localization, and relative and collaborative positioning. This paper provides a framework for modeling the DoA angle in satellite communications with respect to the elevation angle. The proposed approach employs a closed-form expression that incorporates various factors, such as the antenna boresight angle, satellite and Earth station positions, and the altitude parameters of the satellite stations. By leveraging this formulation, the work accurately calculates the Earth station’s elevation angle and effectively models the DoA angle. To the authors’ knowledge, this contribution is unique and has not been previously addressed in the available literature. Furthermore, this paper studies the impact of spatial correlation in the channel on well-known DoA estimation techniques. As a significant part of this contribution, the authors introduce a signal model incorporating correlation in satellite communication. Although selected studies have presented spatial signal correlation models in satellite communications to analyze the performance metrics, such as the bit error or symbol error probability, outage probability, and ergodic capacity, this work stands out by presenting and adapting a correlation model in the signal specifically for studying DoA estimations. Accordingly, this paper evaluates DoA estimation performance using root mean square error (RMSE) measurements for different satellite communication link conditions (uplink and downlink) through extensive Monte Carlo simulations. The simulation’s performance is evaluated by comparing it with the Cramer–Rao lower bound (CRLB) performance metric under additive white Gaussian noise (AWGN) conditions, i.e., thermal noise. The simulation results demonstrate that incorporating a spatial signal correlation model for DoA estimations significantly improves RMSE performance in satellite systems.

## 1. Introduction

Accurate estimation of direction-of-arrival (DoA) is a constantly evolving research area with broad applications in multiple communication technologies, including but not limited to radar, sonar, cellular, geophysics, acoustic tracking, and astronomy. These applications span numerous fields, such as military, satellite, vehicular, and more [1]. In satellite communication systems, the DoA-based measurements for locating objects of interest, such as ground signal sources or vice versa, have become increasingly important in recent years [2]. The communication between ground and space segments in satellite systems is established through basic parameters, such as frequencies and orbits [3]. In general, the satellite orbits are ellipses within the orbital plane, but, in the case of zero eccentricity, they are considered circular orbits [3]. Satellite altitude can vary significantly, from low Earth orbits (LEO) at heights of 180–2000 km to geostationary Earth orbits (GEO) at approximately 35,786 km above the Earth’s surface [4].

DoA estimation methods have been extensively researched for satellite communication systems, as these methods have unique strengths and weaknesses concerning their techniques, speed, computational complexities, accuracy, and channel characteristics [5]. Consequently, DoA estimation methods find a wide range of applications in satellite communication systems, such as altitude determination [6], geolocation accuracy [7,8], estimation accuracy [9], target localization [10], relative positioning [11], and collaborative positioning [12]. To enhance the DoA estimation performance and minimize errors, which, in turn, improves the communication system’s performance and accuracy, it is crucial to model the DoA angle and satellite channel accurately.

This paper provides a framework for modeling the DoA angle in satellite communications with respect to the elevation angle. The proposed approach is novel as it employs a closed-form expression utilizing the geographic coordinate system (i.e., latitude and longitude of the Earth station) and the satellite stations’ altitude parameters and antenna boresight angle information to calculate the Earth station’s elevation angle and accurately model the DoA angle. To the authors’ knowledge, this contribution is unique as no other works are present in the literature modeling the DoA with respect to the elevation angle, i.e., a crucial parameter in satellite communication. Thus, it serves as an advantage, allowing the authors to study the performance of DoA techniques, especially at higher DoA angles, such as closer to −(π/2) and (π/2), with respect to the antenna boresight and elevation angle.

Furthermore, this paper studies the impact of spatial correlation in the channel on well-known DoA estimation techniques via comprehensive comparative analysis. As a significant part of this contribution, the authors introduce a signal model incorporating correlation in satellite communication. Some studies have presented spatial signal correlation models in satellite communications to analyze the performance metrics, such as the bit error or symbol error probability, outage probability, ergodic capacity, etc. However, this work presents and adapts a spatial signal correlation model for studying DoA estimations. The estimation performance evaluation considers using root mean square error (RMSE) measurements for different satellite communication link conditions through extensive Monte Carlo simulations.

The paper is organized as follows: Section 2 reviews the challenges in DoA estimation methods for satellite communication and addresses the importance of an accurate satellite channel model. Section 3 considers the satellite geometries for DoA estimation in both uplink and downlink scenarios for two different satellite systems, i.e., GEO and LEO satellites. For uplink communication, the study utilized spherical geometries to determine the satellite’s look angles and range, whereas for downlink communication, basic geometries were employed for satellites. Section 4 presents the signal model for both uplink and downlink scenarios for the aforementioned satellite systems. Notably, for LEO satellite systems, additional parameters, such as the satellite’s altitude, velocity, and Doppler shift (due to the satellite’s motion), were taken into account. In Section 5, two DoA estimation techniques are outlined: the classical delay and sum (DAS) method and subspace-based multiple signal classification (MUSIC) algorithms [5]. The aim is to assess the ability of different DoA estimation techniques to accurately estimate the DoA in a multiple-antenna-based single receiver system while operating under diverse channel impairments. The impairments that are examined in this paper comprise additive white Gaussian noise (AWGN), i.e., thermal noise, Rician fading [13] with channel-phase random variables, and spatial correlation in multi-antennas [14]. The efficacy of the proposed technique is evaluated through comprehensive Monte Carlo simulations in MATLAB. To validate the precision of the simulations, Section 6 presents the Cramer–Rao lower bound (CRLB) as a performance measure for the AWGN scenario. Section 7 provides a detailed analysis of the numerical outcomes. Finally, Section 8 summarizes the key findings of this study.

## 2. Related Work

This section explores the existing literature on DoA estimation methods in the context of satellite communication systems, considering various factors that may affect efficient communication. It also discusses the crucial aspects of accurately modeling the satellite channel and compares the findings of this study with the literature.

The estimation of DoA is a topic of considerable interest in the field of satellite communication systems. Its investigation has been extensive, with researchers exploring a variety of scenarios and confronting a multitude of practical challenges. Among these challenges are accurate angle of arrival (AoA) estimation, optimization of positioning accuracy in the presence of system failures, resolution of phase measurement ambiguities, and geolocation accuracy for moving objects. Each of these challenges has been tackled by researchers, who have provided valuable insights and potential solutions. For instance, in [9], the researchers introduced a method for precisely estimating the AoA of wideband signals that impinge on localized hybrid antenna arrays mounted on satellites. To tackle system failures, the authors in [6] performed a novel satellite altitude determination method using the DoA estimation of a ground source for three-axis stabilized communication satellites. To optimize the positioning accuracy of satellites, the authors in [8] utilized receiver antenna beamforming and improved receiver sensitivity. Consequently, to improve the ambiguity resolution of phase measurements and positioning accuracy with AoA estimation, the authors in [11] deployed instruments across multiple flying satellites and performed data link synchronization and channel estimation. In addition, to address issues with mobile objects that affect the accuracy of location, the authors of [7] studied two nonlinear optimization (NLO) algorithms, namely, velocity and time delay, which enhance the geolocation accuracy and confidence intervals for moving objects. The challenges are broader than the above discussions. For instance, the outdoor scenarios surpluses complexity was addressed by the authors in [12] through AoA-assisted GNSS collaborative positioning that reduced computational complexity and improved the accuracy of collaborative positioning and AoA measurements. Appropriate geometry selection for target localization problems is also a big challenge, as mentioned in [10], so the authors employed triangulation using a spherical Earth model and a satellite observer over the spherical surface utilizing spherical trigonometry and singular value decomposition (SVD).

The literature review recognized that the DoA estimation method is challenging in satellite communication systems due to the numerous factors involved in establishing effective communication. It also requires accurate channel-phase estimation. The problem of phase estimation has been studied extensively in various contexts. An example is provided in [15], where the authors conducted a statistical analysis to detect bias problems in phase tracking and provided solutions for correcting these biases.

Furthermore, the literature review identified spatial correlation in multiple input multiple output (MIMO) communication channels as a prevalent issue arising from channel properties, antenna patterns, or inadequate spacing between the antennas at the transmitter or the receiver. In general, the influence of spatial correlation on system performance has been widely studied under different contexts. The authors in [16] considered whitening transformations at both the transmitter and the receiver and eliminated spatial correlations to enhance the error performance of LEO Satcom systems based on MIMO-orthogonal time frequency space (OTFS). Consequently, the authors in [17] quantified the effects of fading correlations in multi-element antenna (MEA) systems using a spatial Rayleigh-fading correlation model (MIMO)-based receiver diversity system. The authors in [18] utilized the formula of the spatial correlation coefficient by combining (i) the characteristics of a multi-beam satellite channel and (ii) the scatter distribution to set up a realistic random channel model and addressed the distribution of scatterers near the receiver antenna in mobile-satellite communication systems, which causes the multipath signals to correlate. Last, but not least, the authors in [19] derived a theoretical formulation to clarify the correlation characteristics differences in different fading settings to fade space diversity and adaptive array antennas. It is important to note that satellite communication operating at higher frequencies (i.e., above 10 GHz) is typically line-of-sight, as there are usually no nearby scatterers at the satellite or the Earth station to create multipath and, thus, achieve fully independent paths. As a result, the authors in [20] reported that they used the downlink channel as a reference for the transmit and receive spatial correlation, with the former referring to the space segment and the latter referring to the system’s ground segment.

The comprehensive literature review thus recognised the critical significance of incorporating spatial correlation in the signal model for satellite communication, which is often overlooked in simulations and analyses for simplicity. Despite its inherent complexity, considering spatial correlation is crucial as it enables the accurate capture of the real-world behavior of satellite communication systems, leading to more realistic and reliable DoA estimation results.

### Motivation and Contribution

Most of the work presented in the literature using spatial signal correlation models in satellite communication revolves around performance investigations of communications in terms of bit error or symbol error probability, outage probability, etc. To the authors’ knowledge, a framework for DoA modeling with respect to the elevation angle and using a spatial signal correlation model to analyze the performance of DoA techniques in satellite communication, especially at higher DoA angles with respect to the antenna boresight and elevation angle, is unique and has not been previously addressed in the available literature. Based on the knowledge gained from the literature review, this work considered a geographic coordinate system for the uplink, enabling a precise representation of locations and a simplified geometry for effective modeling of the downlink. However, the focus of this work leans towards the downlink as environmental influences, i.e., fading and correlation, are typically present only in the downlink because of the terrain and infrastructure in the receiver environment. In contrast, the uplink lacks such environmental influences, resulting in a scenario where only additive white Gaussian noise (AWGN) can be considered, thus indicating only one result for the uplink. Similarly, due to the nature of free space in the uplink, the concept of correlation is not applicable. In this scenario, the signals are inherently fully correlated in space, meaning they remain 100% correlated at all times.

## 3. Satellite Geometries

### 3.1. Uplink Satellite Geometry for DoA Estimation

**Figure 1:**
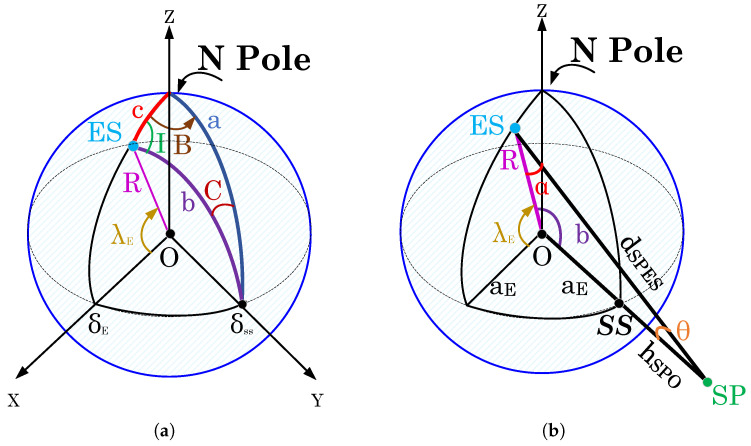
Uplink satellite geometry for DoA estimation using a geographic (i.e., spherical) coordinate system: (**a**) spherical triangle with angles “abc” as arcs of great circles, (**b**) plane triangle “OESSP” to determine the elevation angle (El) of the Earth station (ES) and slant distance ($d_{\mathrm{SPES}}$) for different satellite systems.

This paper considers different satellite systems, i.e., GEO and LEO satellites. This section first discusses the uplink system geometry of the GEO satellite system and how it is modified for use in the LEO satellite system by adjusting the relevant parameters. The illustration shown in Figure 1 depicts the spherical geometric approximation utilized for the transmitting antenna of the Earth station, which is situated on the Earth’s surface and communicates with the GEO satellite station equipped with receiving antennas during uplink communication. The GEO satellite station is positioned to travel eastward at the same rotational speed as the Earth, maintaining a stationary position relative to the Earth. The satellite station has a circular orbit and zero inclination [2]. The Earth station is labeled as ES, the satellite station is labeled as SP, and the center of the Earth is indicated by the symbol O. The azimuth and elevation angles of the ES are denoted as $A_{z}$ and El, respectively. The $A_{z}$ is the horizontal angle from true north in a clockwise direction of the satellite with a range of (0 to 360)${}^{\circ }$. On the other hand, El is the angle between the satellite and the observer’s horizon plane and has a range of (0 to 90)${}^{\circ }$ [3]. The ES is defined by its geographic coordinate system (GCS) (i.e., a spherical coordinate system), denoted as $\left(\delta_{E},\lambda_{E}\right)$, where $\delta_{E}$ is the longitude measured in the positive east direction, and $\lambda_{E}$ is the latitude of the ES measured in the positive north direction. The sub-satellite point is denoted as SS, and its longitude is represented by $\delta_{ss}$ and its latitude by $\lambda_{ss}$. The latitude of the sub-satellite point is ideally $0^{\circ }$, as it is positioned directly above the equator. The slant distance from the ES to the SP is denoted as $d_{\mathrm{SPES}}$, while α is the critical angle that needs to be determined [2,21].

Figure 1a shows a spherical triangle with angles “abc” as arcs of great circles, while Figure 1b demonstrates a plane triangle “OESSP”. The angle I shown in Figure 1a, which is the angle between the plane containing c and the plane containing b, is commonly employed to calculate the $A_{z}$ angle [2]. Our model focused only on determining El. Thus, calculations related to $A_{z}$ are not included, and are assumed to be 0${}^{\circ }$. Thus, the information is summarized as follows: (i) $a=90^{\circ }$, the angle between the distribution for the range to the north pole and the radius to the SS (i.e., sub-satellite point), (ii) $c=90^{\circ }-\lambda_{E}$, the angle between the radius to the ES and the radius to the north pole, and (iii) $B=\delta_{E}-\delta_{SS}$, the angle between the plane having c and the plane containing a. Therefore, the angle b between the radius of ES and the radius of SS is determined using Napier’s rules as below:

(1) $$b=\arccos\left(\cos B\cos\lambda_{E}\right),$$

where B is negative when ES is west of the sub-satellite point and positive when east. To calculate El, the sine rule for plane triangles is applied to the triangle shown in Figure 1b [2] as below:

(2) $$\mathrm{El}=\arccos\left(\frac{a_{\mathrm{SPO}}}{d_{\mathrm{SPES}}}\sin b\right),$$

In Equation (2), $a_{\mathrm{SPO}}$ is the distance from the SP to O and is given by:

(3) $$a_{\mathrm{SPO}}=a_{E}+h_{\mathrm{SPO}}$$

where $a_{E}$ represents the equatorial radius of the Earth, typically considered to be 6378 km. In general, the average radius of the Earth (R) is 6371 km. Thus, $(a_{E}$ ≈ R). Moreover, the altitude for a GEO satellite is denoted as $h_{\mathrm{SPO}}$, which is 35,786 km. Thus, the value of $a_{\mathrm{SPO}}$ sums to 42,164 km. Therefore, $d_{\mathrm{SPES}}$ in Equation (2), is determined by applying the cosine rule for plane triangles to the triangle of Figure 1b as below:

(4) $$d_{\mathrm{SPES}}=\sqrt{R^{2}+a_{\mathrm{SPO}}^{2}+2Ra_{\mathrm{SPO}}\cos b}\;,$$

Finally, the angle θ in Figure 1b is given by:

(5) $$\theta =180^{\circ }-\left(\alpha +b\right),$$

where angle $\alpha =90^{\circ }+\mathrm{El}$, and θ is the DoA of the GEO satellite.

**Figure 2:**
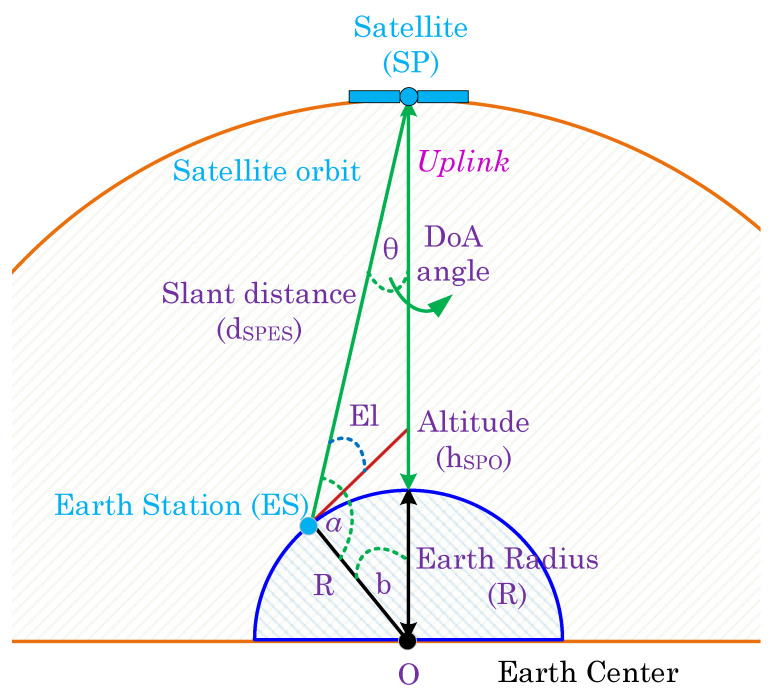
Uplink satellite geometry for DoA estimation.

For the uplink communication of a LEO satellite, the same geometric configurations as those used for a GEO satellite, shown in Figure 1, is applied. To determine the El, $d_{\mathrm{SPES}}$, and θ of the LEO satellite system, Equations (1)–(5) are employed. However, as the altitude of the LEO satellite system ranges from 180–2000 km, the calculation of $d_{\mathrm{SPES}}$ for the LEO satellite system requires a different distance value, $a_{\mathrm{SPO}}$, from SP to O, compared to the GEO satellite system. Thus, $a_{\mathrm{SPO}}$ is set to 1500 km for the LEO satellite system. The illustration of the uplink satellite geometry for DoA estimation in different satellites is shown in Figure 2.

### 3.2. Downlink Satellite Geometry for DoA Estimation

**Figure 3:**
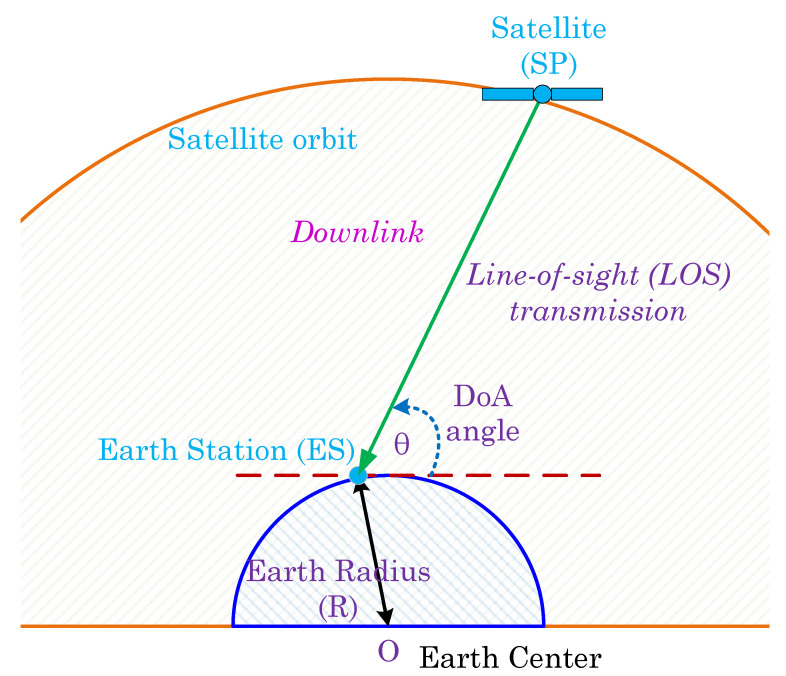
Downlink satellite geometry for DoA estimation.

In the downlink signal model shown in Figure 3, the angle between two points, represented by their respective coordinates, is calculated using the inverse tangent function. More precisely, when the transmitting satellite SP is located at coordinates $\left(X_{\mathrm{Tx}},Y_{\mathrm{Tx}}\right)$ and the receiving ES is at coordinates $\left(X_{\mathrm{Rx}},Y_{\mathrm{Rx}}\right)$ in the Earth-centered inertial (ECI) coordinate system, the angle between them can be computed as follows:

(6) $$\theta =\arctan\left[\left(Y_{\mathrm{Rx}}-Y_{\mathrm{Tx}}\right)/\left(X_{\mathrm{Rx}}-X_{\mathrm{Tx}}\right))\right].$$

Therefore, θ is the downlink DoA angle at the ES, i.e., above the Earth’s surface around the horizon line. Thus, for simplicity, the DoA angle is represented as θ for both uplink and downlink scenarios.

## 4. Signal Models

### 4.1. Uplink Signal Model for Different Satellite Systems

**Figure 4:**
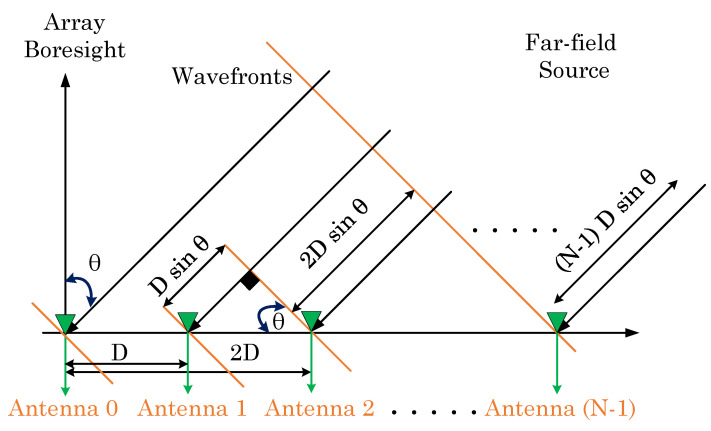
Uniform linear array geometry at satellite systems.

This section presents the signal model for the uplink communication in different satellite systems, i.e., the GEO and LEO satellites in Figure 4. The signal model incorporates a receiver with a uniform linear array (ULA) geometry comprising *N* directional antenna elements, capable of receiving signals from a source in the far field. The computation of the time delay of arrival between the signals received at antenna element 0 and antenna element *n*∈[0,⋯,N−1] in a multi-antenna system is based on a careful analysis of the system geometry and application of fundamental trigonometric principles. The time delay of arrival is mathematically expressed as $\Delta t_{n}$ and given by the following expression:

(7) $$\Delta t_{n}=\frac{nD\sin\theta }{c};\theta \in \left[-90^{\circ },90^{\circ }\right],$$

Equation (7) highlights the significance of the distance *D* between the antenna elements and the speed of light c for receiving signals accurately. To prevent aliasing in space, *D* is conventionally set to 0.5 $\lambda_{c}$. The wavelength of the propagating wave is represented by $\lambda_{c}=c/f_{c}$, where $f_{c}$ signifies the frequency. Furthermore, the angle θ reflects the direction of the transmitter, which provides crucial information about the received signal. Therefore, the received signal r can be represented mathematically as below:

(8) r=AHs+v,

The signal vector s, with dimensions 1×ξ, can be expressed as $s=[s_{0},s_{1},\cdots s_{\left(\xi -1\right)}]$, where ξ represents the number of samples. The noise vector v is an [N×ξ] matrix given by:

(9) $$v={\left[v_{0},v_{1},\cdots v_{n},\cdots v_{\left(N-1\right)}\right]}^{T},$$

In Equation (9), $v_{n}$ is a 1×ξ row vector representing the noise at the *n*th element over the ξ samples as below:

(10) $$v_{n}=\left[v_{n0},v_{n1},\cdots ,v_{ni},\cdots ,v_{n(\xi -1)}\right],$$

The variable $v_{ni}$ represents the complex zero-mean AWGN [13] at the receiver, with a noise power of $\sigma_{v}^{2}$ for the *i*th sample of noise for the *n*th antenna element. The signal-to-noise ratio (SNR) is denoted as γ in this paper, with the signal power represented as $E{\left[s\right]}^{2}$, as given by the following mathematical expression:

(11) $$\gamma =E{\left[s\right]}^{2}/\sigma_{v}^{2}.$$

Finally, from Equation (8), the steer vector component A, with dimension N×1, is given by:

(12) $$A={\left[a_{0},a_{1},\cdots a_{n},\cdots a_{\left(N-1\right)}\right]}^{T},$$

where $a_{n}$ from Equation (12) is first described for a LEO satellite as follows:

(13) $$a_{n}=\exp\left(-j2\pi n\frac{D}{\lambda_{d}}\sin\theta +j\phi_{n}\right);\forall n,$$

Thus, ignoring subscript *n* in the notation, ϕ denotes the channel-phase of the receiver system. The phase is considered to be a random variable and has a uniform distribution within the range of (−π<ϕ<π). Moreover, the variable $\lambda_{d}$ is defined as $\lambda_{d}=f_{c}+f_{d}$. Note that $f_{d}$ is the Doppler shift [22] considered in the LEO satellite system geometry. This effect is caused by the relative motion between the satellite and the Earth station on the ground given by:

(14) $$f_{d}=\frac{V_{\mathrm{sat}}}{\lambda_{c}}\cos\varphi ,$$

In Equation (14), φ is the angle of the velocity vector, i.e., the driving direction concerning the satellite. It is a randomly distributed value between 0 and π. However, this work assumes φ=0, generating a maximum value of $f_{d}$ among values for different angles. Notably, the Doppler model used in this paper pertains to a stationary (or slowly moving) target, where the ground velocity is considerably lower than the satellite’s motion concerning the Earth. Thus, the LEO satellite is considered to be moving along the orbit with a velocity $V_{\mathrm{sat}}$, while the ES is static. Note that when $f_{d}=0$, then, Equation (13) can be defined as the steer vector component $a_{n}$ for a GEO satellite. It is essential to note that, in the uplink scenario, the channel matrix H is set to unity, as the uplink transmission lacks environmental influences due to the terrain and infrastructure in the receiver environment. Therefore, the multiplication involving A, H, and s is carried out through matrix multiplication, resulting in a [N×ξ] matrix. Consequently, due to this configuration, the received signal r is also a [N×ξ] matrix. Accordingly, the received signal in the uplink scenario can be characterized as an AWGN channel with a steering vector.

### 4.2. Downlink Signal Model for Different Satellite Systems

The received signal in the downlink scenario is constructed similarly to the uplink scenario, consisting of the signal vector s, noise vector v, steer vector A, and channel vector H, as presented in Equation (8). However, a multipath environment with Rician fading is considered in the downlink scenario. Hence, unlike in the uplink scenario, the channel vector [23] is not set to unity. The ULA geometry in Figure 4 only illustrates the direct paths for better visualization and does not include indirect paths, such as diffracted or scattered paths. Moreover, the indirect paths are assumed to be only reflected paths to simplify the model. Thus, the expression for H is given as follows:

(15) $$H=[h_{0},h_{1},\cdots h_{n},\cdots h_{\left(N-1\right)}],$$

The component $h_{n}$ includes the Rician fading, which is expressed as:

(16) $$h_{n}=\sum_{m=1}^{M}g_{\left(n,m\right)},$$

where *M* indicates the number of paths. Thus, as per the model, when m=1, the direct path is obtained. Consequently, the transmitter’s direction θ from Equation (13) for the direct path in the downlink scenario is considered as $\theta_{1}$, i.e., $\theta =\theta_{1}$. Furthermore, this paper accounted for a normalized Rician channel with two paths (M=2). Therefore, $\theta_{2}$ is the angle of the reflected path, generated randomly. Thus, the channel component reduces to the below:

(17) $$h_{n}=g_{\left(n,1\right)}+g_{\left(n,2\right)},$$

The channel coefficients for the line-of-sight (LOS) and non-line-of-sight (NLOS) paths are denoted by $g_{\left(n,1\right)}$ and $g_{\left(n,2\right)}$, respectively. The LOS path is assumed to have a fixed coefficient of 1, while the NLOS path coefficient $g_{\left(n,2\right)}$ is expressed as follows:

(18) $$g_{\left(n,2\right)}=\sqrt{\left(\omega_{1}^{2}+\omega_{2}^{2}\right)},$$

The parameters $\omega_{1}$ and $\omega_{2}$ are two independent Gaussian random variables with a variance of $\sigma_{R}^{2}/\left(2K\right)$ each, where $\sigma_{R}^{2}$ equals 1, and *K* represents the Rice factor value [13], which is expressed as below:

(19) $$K=\frac{{\left|g_{\left(n,1\right)}\right|}^{2}}{{\left|g_{\left(n,2\right)}\right|}^{2}}.$$

### 4.3. Spatial Signal Correlation Model for Satellite Systems

In the received signal model, the final step involves introducing a signal model with correlation in satellite communication. It should be noted that correlation considerations are typically unnecessary in uplink transmission due to the lack of environmental influences and the characteristics of free space. However, for the downlink scenario, incorporating correlation becomes essential. This work considered a carrier frequency of 28 GHz, which falls within the Ka-band satellite communication. At such a high frequency, the nature of antenna systems is highly directional. Thus, a spatial signal correlation model that considers the antenna beam’s directionality is necessary to model the wireless channel accurately. Therefore, the authors utilized a spatial correlation model discussed in their previous work [14], which characterizes the channel by a correlation matrix that varies with the direction of transmission and reception. Thus, the channel is transformed to a spatially correlated Rician channel at the receiver for the downlink scenario. In directional spatial correlation models, the decorrelation distance value is typically small, often measured in meters or less.

## 5. DoA Estimation Techniques

### 5.1. Delay and Sum (DAS) Technique

In the delay-and-sum (DAS) method for estimating DoA, the power of the signal is computed for each angle, and the direction with the highest power is selected [5]. The DAS beamformer output for various satellites is expressed as follows:

(20) $$P_{DAS}\left(\theta \right)=A^{H}Cov_{D}\left(r\right)A,$$

where $Cov_{D}$ is the spatial covariance matrix for ξ number of datas (i.e., samples) collected from the antennas and is given by:

(21) $$Cov_{D}\left(r\right)=\frac{1}{\xi }\sum_{i=0}^{\xi -1}r_{i}r_{i}^{H},$$

where ${}^{H}$ represents the Hermitian matrix.

### 5.2. Multiple Signal Classification (MUSIC) Algorithm

To detect frequencies in a signal, the multiple signal classification algorithm (MUSIC) performs an eigen decomposition on the covariance matrix of the received signal vector r [5,24]. The process utilizes the correlation matrix $\sigma_{v}^{2}I$, where, I is the identity matrix and, therefore, expressed as below:

(22) $$Cov_{M}\left(r\right)=A\;E\left[ss^{H}\right]\;A^{H}+\sigma_{v}^{2}I,$$

In Equation (22), the eigenvectors of the covariance matrix define the signal subspace, which is represented by the column space of matrix A, and the noise subspace, represented by matrix $V_{n}$. The estimator function takes advantage of the orthogonality between the signal and noise subspaces, and has peaks that correspond to the DoA of each signal, as expressed below:

(23) $$P_{MUSIC}\left(\theta \right)=\frac{1}{A^{H}V_{n}V_{n}^{H}A},$$

where $P_{MUSIC}\left(\theta \right)$ obtains a significant value when the value of angle θ (i.e., part of matrix A) corresponds to the DoA of one of the signals.

## 6. Cramer–Rao Lower Bound (CRLB)

In this section, a performance evaluation of a parameter estimation technique was conducted by considering a set of performance bounds [25]. These bounds were employed to evaluate the possible performance of the technique. Therefore, the Cramer–Rao lower bound (CRLB), which provides a lower bound on the estimation variance of an unbiased estimator for the parameter θ, is denoted as $\sigma_{\mathrm{CR}}\left(\theta \right)$. The expression for CRLB is as follows:

(24) $$\sigma_{\mathrm{CR}}\left(\theta \right)=\sqrt{\frac{1}{\xi }\left[\frac{1}{\left(N\gamma \right)}+\frac{1}{{\left(N\gamma \right)}^{2}}\right]\frac{6}{\left(N^{2}-1\right)}},$$

Thus, Equation (24) expresses $\sigma_{\mathrm{CR}}\left(\theta \right)$ for DoA estimation as a function of *N*, ξ, and γ, thereby simplifying its derivation. However, the model for $\sigma_{\mathrm{CR}}\left(\theta \right)$ is only applicable to cases where AWGN, or thermal noise, is present. The calculation of this model does not account for the random variable of the channel phase.

## 7. Numerical Analysis

### 7.1. Performance Evaluation of DoA Estimation Techniques

The performance of DoA estimation is assessed using a simulation environment in MATLAB and is measured in terms of the root mean square error (RMSE), as discussed in [1]. The expression for RMSE is as follows:

(25) $$\sigma \left(\theta \right)=\sqrt{E{\left[\left(\hat{\theta }\right)-\left(\theta \right)\right]}^{2}},$$

where $\hat{\theta }$ and θ represent the estimated and actual values of the DoAs, respectively. Thus, the RMSE performance is measured in degrees, denoted as σ(θ), plotted against the γ, i.e., the signal-to-noise ratio in decibels [dB].

**Figure 5:**
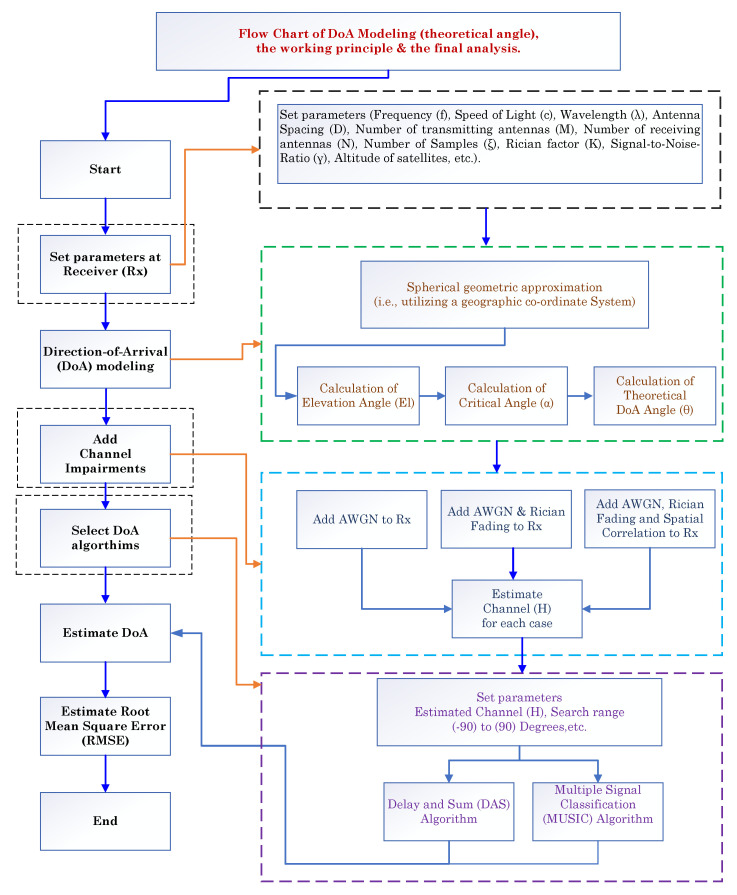
Flowchart demonstrating the proposed model’s complete analysis and working principle.

The simulation parameters for the signal model and the notations used for the uplink and downlink scenarios are specified in Table 1. Unless explicitly stated otherwise, the values specified in the table are used in this study. A flowchart illustrating the proposed model’s complete analysis and working principle is presented in Figure 5. The flowchart serves as a visual aid of the overall process and enables clear comprehension of how the proposed model functions. The RMSE for the DAS and MUSIC methods were calculated to evaluate their performance under various channel conditions and diverse scenarios. The following channel conditions were considered: (i) AWGN only, (ii) AWGN and Rician fading, and (iii) spatially correlated channels with AWGN and Rician fading. The investigations were treated as separate cases, allowing for a comprehensive analysis of the method’s performance under different conditions.

#### Results

**Figure 6:**
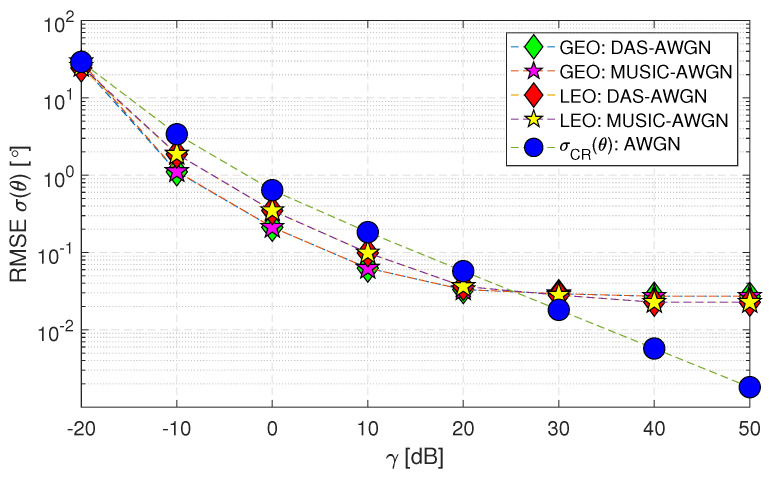
RMSE σ(θ) performance of uplink geostationary and low Earth orbit satellites using DAS and MUSIC techniques for AWGN only (Case-i(a)).

**Figure 7:**
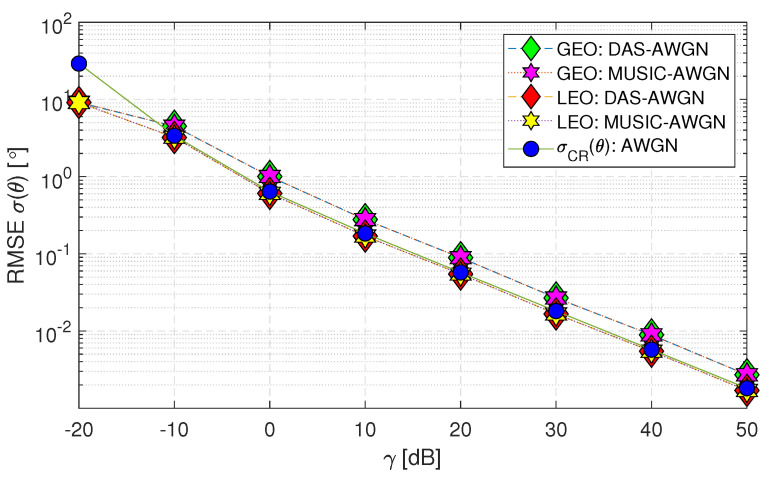
RMSE σ(θ) performance of downlink geostationary and low Earth orbit satellites using DAS and MUSIC techniques for AWGN only (Case-i(b)).

The uplink and downlink scenarios with AWGN are considered as (Case-i(a)) and (Case-i(b)), denoted as “DAS-AWGN” and “MUSIC-AWGN” for different satellites, i.e., GEO and LEO satellites in Figure 6 and Figure 7. The uplink transmission in satellite communication, lacking significant environmental influences and channel H set to unity, simplifies to an AWGN scenario, as shown in Figure 6. Thus, the uplink analysis typically produces a single result, as other factors, such as fading or correlation, are absent. Therefore, based on the elevation angle range of (0 to 90) ${}^{\left[\circ \right]}$, the maximum DoA angles for uplink GEO and LEO satellites are 8.5671 and 52.8727 ${}^{\left[\circ \right]}$, respectively. Therefore, at this range, the RMSE σ(θ) performance for higher SNR values has lower errors, specifically from 30 [dB]. In comparison, the estimation error is high at lower SNR values.

Note that, from Figure 7 onwards, all the outputs achieved are only for the downlink geometry under different cases. Therefore, the downlink scenario with AWGN for GEO and LEO satellites in Figure 7 (Case-i(b)) depicts the observations below:

- The DAS and MUSIC methods are considered optimal when assuming AWGN (i.e., matched filtering provides the best results for a predefined noise distribution).
- The RMSE performance of both DAS and MUSIC methods for the LEO satellite matches the $\sigma_{\mathrm{CR}}\left(\theta \right)$ within the range of (−10 to 50) [dB]. However, for the GEO satellite, the performance is close to $\sigma_{\mathrm{CR}}\left(\theta \right)$.
- When the range of γ decreases below −10 [dB], the DAS and MUSIC curves deviate beyond the Cramer–Rao lower bound ($\sigma_{\mathrm{CR}}\left(\theta \right)$) for both GEO and LEO satellites. This deviation occurs because the estimation of the channel-phase random variable is limited to a range of (−π<ϕ<π). Consequently, the standard deviation of the ϕ analysis is constrained to this range and remains constant when the γ value drops to −20 [dB]. In contrast, the $\sigma_{\mathrm{CR}}\left(\theta \right)$ applies to all values and can take any real numbers.

**Figure 8:**
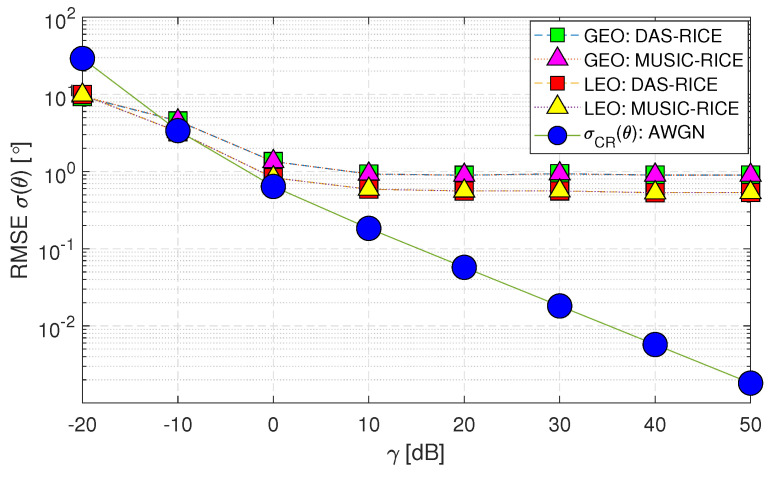
RMSE σ(θ) performance of downlink geostationary and low Earth orbit satellites using DAS and MUSIC techniques for AWGN and Rician fading scenario (Case-ii).

Figure 8 (Case-ii) demonstrates the impact of AWGN and Rician fading, where the channel phase is modeled as a random variable. The performance of the DoA estimation methods for both GEO and LEO satellites is evaluated and denoted as “DAS-RICE” and “MUSIC-RICE”. Notably, the RMSE statistics σ(θ) exhibit a decline from 0 [dB] onwards for (Case-ii), indicating the adverse effects of a multipath environment on the system model. As a result, the performance of the DoA estimation methods deteriorates under these channel conditions.

**Figure 9:**
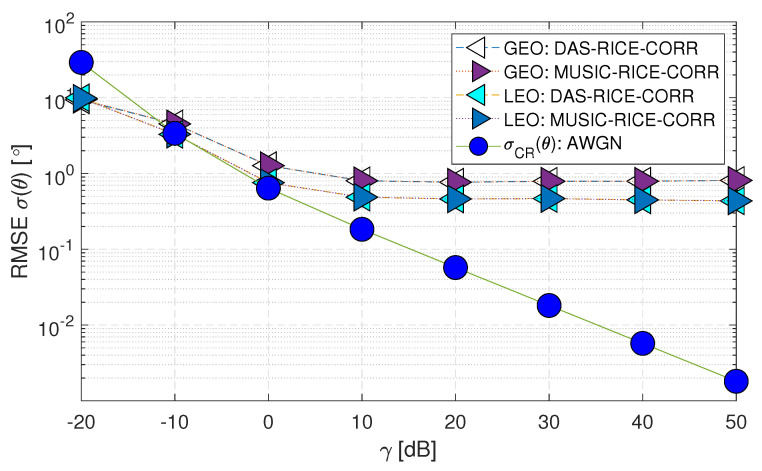
RMSE σ(θ) performance of downlink geostationary and low Earth orbit satellites using the DAS and MUSIC techniques for spatially correlated channels with AWGN and Rician fading (Case-iii).

**Figure 10:**
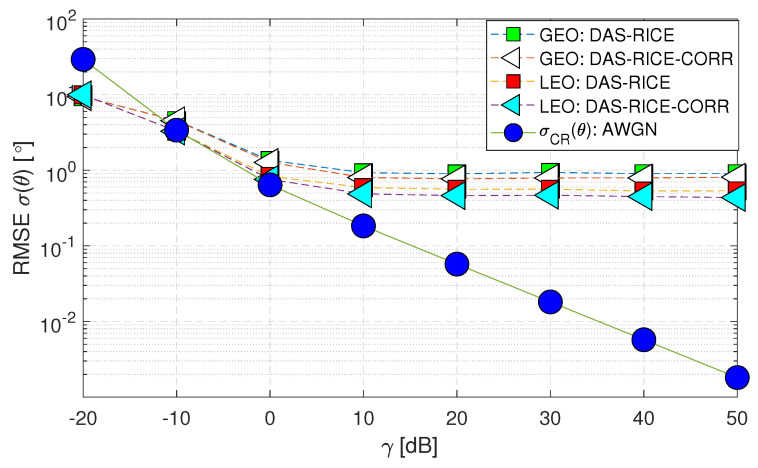
RMSE σ(θ) performance of downlink geostationary and low Earth orbit satellites using the DAS technique comparing (Case-ii) with (Case-iii).

Figure 9 illustrates the performance of DoA estimation techniques for GEO and LEO satellites under the scenario where spatial correlation is present in addition to AWGN and Rician fading. The figures denote the techniques as “DAS-RICE-CORR” and “MUSIC-RICE-CORR” for DAS and MUSIC methods, respectively. It can be observed that the performance of (Case-iii) shown in Figure 9 appears similar to that of (Case-ii) presented in Figure 8. For precise visualization of the graphs and to study the effect of spatial correlation in a multipath environment, the AWGN and MUSIC methods were excluded; the comparison between (Case-ii) and (Case-iii) is presented in Figure 10.

Figure 10 illustrates that the performances of (Case-ii) and (Case-iii) are similar for both GEO and LEO satellites. However, (Case-iii) performs slightly better due to the inclusion of spatial correlation. The reason for this can be explained as follows: since the line-of-sight (LoS) angle is less random, the parameter estimation for it is the same for all paths. This leads to a better performance for a limited number of antennas (N=4), as seen in the “LEO:DAS-RICE-CORR” curve. Therefore, subsequent studies only focus on this curve for a more accurate comparison.

**Figure 11:**
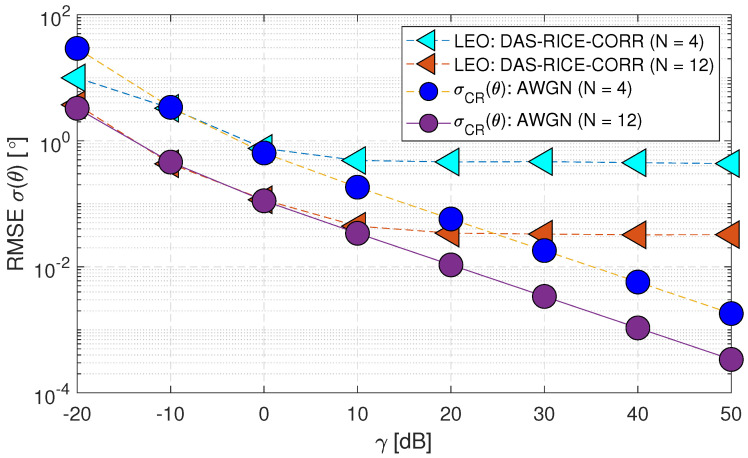
RMSE σ(θ) performance of downlink low Earth orbit satellite using the DAS technique for change in antenna values in spatially correlated channels with AWGN and Rician fading (Case-iv).

Figure 11 presents the performance of the “LEO:DAS-RICE-CORR” case with a higher number of antennas (N=12), denoted as (Case-iv). The results reveal a noteworthy improvement in the RMSE σ(θ) performance for both “LEO:DAS-RICE-CORR” and $\sigma_{\mathrm{CR}}\left(\theta \right)$ cases, which demonstrates the positive impact of a higher number of antennas on the system model.

**Figure 12:**
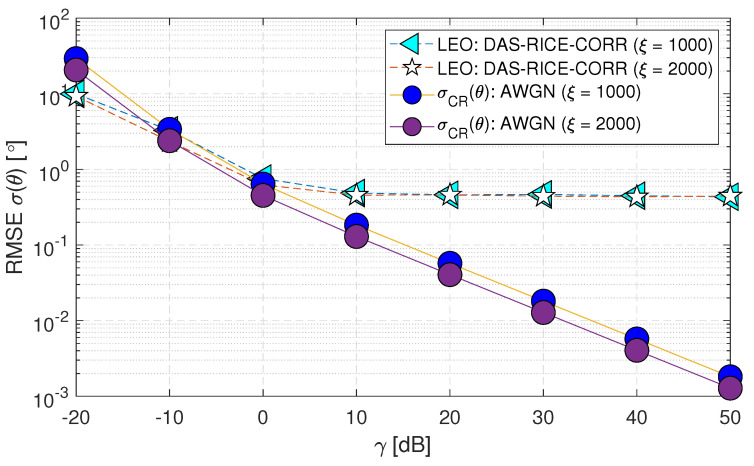
RMSE σ(θ) performance of downlink low Earth orbit satellite using the DAS technique for change in sample sizes in spatially correlated channels with AWGN and Rician fading (Case-v).

Certain parameters are often held constant to maintain consistency and ensure accurate results. In this work, three parameters are considered fixed unless otherwise specified: (i) ξ (i.e., samples) at 1000, (ii) *K* (i.e., K factor value) at 30 [dB] in the Rician fading channel and (iii) $D_{0}$ (i.e., decorrelation distance) at the mobile station (MS) and base station (BS) as 0.5 λ and 13 λ for the prior investigations. However, the subsequent studies deviated from these fixed values in certain case scenarios to understand how these parameters affect the overall results. By varying these parameters, the study identified their individual impact and drew more robust conclusions from their analysis. Firstly, in Figure 12, two ξ values (i.e., 1000 and 2000) are evaluated as (Case-v) that studies the impact of change in sample values on RMSE σ(θ) performance. A little deviation is observed on the “LEO:DAS-RICE-CORR” curve, while in the $\sigma_{\mathrm{CR}}\left(\theta \right)$ case, there is a good improvement for an increase in the ξ value.

**Figure 13:**
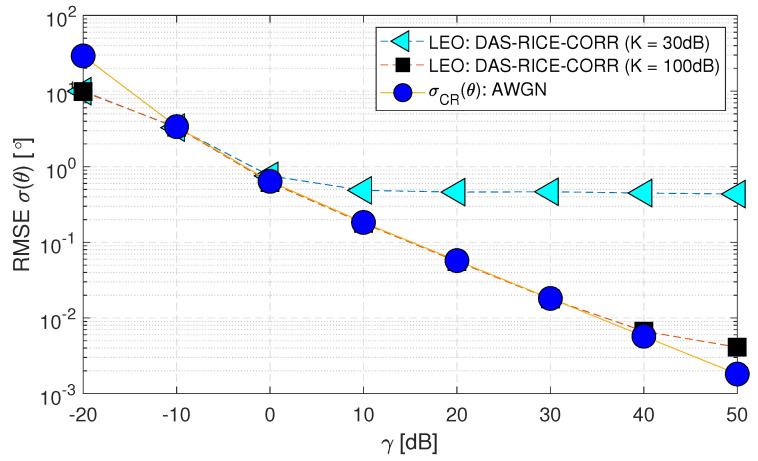
RMSE σ(θ) performance of downlink low Earth orbit satellite using the DAS technique for change in *K* factor values in spatially correlated channels with AWGN and Rician fading (Case-vi).

Similarly, in Figure 13, two *K* factor values (i.e., 30 and 100) [dB] are evaluated as (Case-vi) that study the impact of multipath on the system model. At a Rician fading channel with K=30 [dB], the RMSE σ(θ) consistently demonstrates poor performance due to the absence of a line-of-sight (LOS) path. However, for K=100 [dB], the RMSE σ(θ) performance is comparable to $\sigma_{\mathrm{CR}}\left(\theta \right)$, and the channel behavior resembles a Gaussian channel having a strong LOS path.

**Figure 14:**
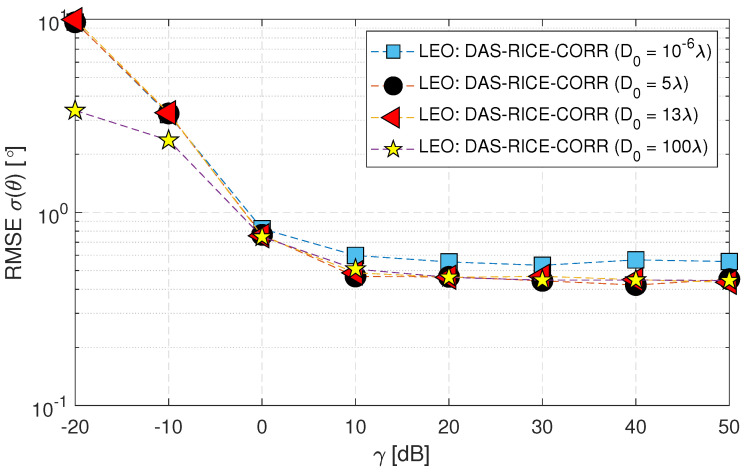
RMSE σ(θ) of downlink low Earth orbit satellite using the DAS technique for changes in decorrelation distance values in spatially correlated channels with AWGN and Rician fading (Case-vii).

The final study investigated the impact of the decorrelation distance $D_{0}$ on the RMSE σ(θ) performance, where the $D_{0}$ values at the BS were varied as $10^{-6}$λ, 5 λ, 13 λ and 100 λ, while the $D_{0}$ value at the MS was fixed. Figure 14 illustrates the findings of this study, which reveal a higher error at $10^{-6}$λ. However, as the decorrelation distance value increases, the error reduces gradually. Notably, the response at 13 λ is similar to that observed at 100 λ. Hence, the RMSE σ(θ) performance is significantly influenced by the decorrelation distance, and increasing it can considerably reduce the error.

## 8. Conclusions

Assessing the performance of DoA estimation methods for satellite communication systems is crucial, as it involves various aspects that must be considered. This paper has provided a framework for modeling the DoA angle in satellite communications with respect to the elevation angle. It employed a closed-form expression incorporating the antenna boresight angle, satellite and Earth station positions, and altitude parameters of the satellite stations to model the DoA angle. Furthermore, this work adapted a spatial signal correlation model to analyze the well-known DoA estimations performance for satellite communications. The study considered different geometries for both uplink and downlink scenarios, and the DoA estimation performance was evaluated under each condition, namely, a geographic coordinate system for the uplink and simplified geometries for the downlink. The analysis in this study primarily focused on downlink transmission due to the presence of fading and correlation, which are typically influenced by factors such as the terrain and receiver infrastructure. In contrast, the uplink transmission, lacking similar environmental influences, was simplified to an additive white Gaussian noise (AWGN) scenario. Additionally, the nature of free space in the uplink made correlation considerations unnecessary. Therefore, the study emphasized the downlink analysis while acknowledging the distinct characteristics and limitations of the uplink scenario. The numerical analysis considered a wide range of signal-to-noise ratio (SNR) values, sample sizes, decorrelation distance $D_{0}$, and Rician *K* factor values. The simulation results indicate that the RMSE σ(θ) performance is acceptable for higher SNR (γ) values, specifically from 30 [dB], as the estimation error increases at lower SNR (γ) values for the uplink scenario. The downlink scenario, including spatial correlation, resulted in a better RMSE σ(θ) performance than the uncorrelated case, indicating parameter estimation for the line-of-sight (LoS) angle was more stable for all paths due to the reduced randomness in the correlation case. The outcomes also suggested that the spatially correlated case matched the AWGN case when there was no fading, i.e., a 100% correlation case, for instance, around K = 100 [dB].

## Figures and Tables

**Table 1:** Parameters Table.

Symbol	Parameter	Units
$f_{c}$	Frequency	28 [GHz]
c	Speed of light	3 × $10^{8}$ [m/s]
*N*	Antenna values	4
γ	Signal-to-noise-ratio	(−20 to 50) [dB]
ξ	Number of samples	1000
*K*	Rice factor value	30 [dB]
$D_{0}$	Decorrelation distance	13λ at BS
$\lambda_{E}$	Latitude of Earth station (Uplink)	−${[}^{\circ }]$
$\delta_{E}$	Longitude of Earth station (Uplink)	−${[}^{\circ }]$
El	Elevation angle of satellites (Uplink)	10 ${[}^{\circ }]$
$h_{\mathrm{SPO}}$	Altitude of satellites (Uplink)	35,786 (GEO) and 1500 (LEO) in [km]
$V_{\mathrm{sat}}$	Velocity of satellites	0 (GEO) and 7.11 (LEO) [km/s]
φ	Velocity vector’s angle of satellites	0 (GEO) and 0 (LEO) in ${[}^{\circ }]$
$f_{d}$	Doppler shift of satellites	0 and 663.600 in [kHz]

## Data Availability

Data is contained within the article.
